# CdSe Quantum Dots in Human Models Derived from ALS Patients: Characterization, Nuclear Penetration Studies and Multiplexing

**DOI:** 10.3390/nano11030671

**Published:** 2021-03-09

**Authors:** Carlota Tosat-Bitrián, Alicia Avis-Bodas, Gracia Porras, Daniel Borrego-Hernández, Alberto García-Redondo, Angeles Martín-Requero, Valle Palomo

**Affiliations:** 1 Centro de Investigaciones Biológicas Margarita Salas CSIC, C/Ramiro de Maeztu 9, 28040 Madrid, Spain; carlota.tosat@cib.csic.es (C.T.-B.); aliciaavis97@gmail.com (A.A.-B.); graciapf@cib.csic.es (G.P.); amrequero@cib.csic.es (A.M.-R.); 2 Neurology Department, ALS Unit, CIBERER U-723, Health Research Institute, 28041 Madrid, Spain; dborregohernandez.imas12@h12o.es (D.B.-H.); mito@h12o.es (A.G.-R.); 3 Centro de Investigación Biomédica en Red de Enfermedades Neurodegenerativas (CIBERNED), Instituto de Salud Carlos III, 28031 Madrid, Spain

**Keywords:** quantum dots, human primary cells, ALS, characterization, multiplexing

## Abstract

CdSe quantum dots (QDs) are valuable tools for deciphering molecular mechanisms in cells. Their conjugation with antibodies offers a unique staining source with optimal characteristics, including increased photostability and narrow emission spectra, allowing for improved multiplexing capabilities using a single excitation source. In combination with pathology models derived from patients, they have great potential to contribute to quantitative molecular profiling and promote personalized medicine. However, the commercial availability of diverse CdSe QDs is still limited and characterization techniques must be performed to these materials or the conjugates developed in the lab to assure a proper function and reproducibility. Furthermore, while there is significant data of QDs experiments in cell lines, the literature with primary human cells is scarce, and QD behavior in these systems may be different. Rigorous characterization data of commercially available QDs and their conjugates with biomolecules of interest is needed in order to establish their potential for target labelling and expand their use among research labs. Here we compare the characterization and labelling performance of different QD conjugates in SH-SY5Y cell line, fibroblasts and immortalized lymphocytes derived from amyotrophic lateral sclerosis patients.

## 1. Introduction

CdSe quantum dots (QDs) are semiconductor nanoparticles formed by a Cd/Se core and a Zn/S coating. They have become valuable fluorescent probes for in vitro and in vivo imaging research due to their unique photoluminiscent properties [1]. QDs display large molar extinction coefficients, high stokes shift, size-dependent color tunability, high resistance to photobleaching and an improved photostability. As consequence, they are ideal for multiplexing applications, with a broad absorption spectrum and narrow emission spectra which allows the excitation of different QDs simultaneously from a single excitation source. In order to render QDs biocompatible, different strategies have been used, including QDs surface coating with hydrophilic ligands such as polyethylene glycol (PEG) or by encapsulating them in amphiphilic polymers [2]. Their use as novel fluorescent probes for biological research was first reported in 1998 [3,4] and since then, multiple applications of QDs in the biological and biomedicine fields have been described [5]. Due to their large surface, QDs have been effectively conjugated with different biomolecules of interest including antibodies, proteins, enzymes or DNA, enhancing their use in biological applications [6,7]. Different QDs reagents and probes are commercially available. First, bioconjugates linked to streptavidin or secondary antibodies can be purchased and are ready to use. Secondly, conjugations kits and QDs functionalized with reactive functional groups are also available to customize the QDs probes in the laboratory. Finally, QDs in organic media can also be obtained, and then the capping exchange should be performed. This availability has increased their use as biological tools for different applications, including their use in diagnostics, mainly in cancer, in which they have demonstrated superior capabilities to accurately characterize tumor parameters in frozen tissues or living cells [8,9]. Furthermore, their ability to undergo FRET experiments enable them to serve as powerful tools in diagnostics [10]. Finally, the combination of these experiments with the use of smartphones for analysis widens their applicability scope [11]. In fact, given their optimal characteristics to serve as sensors their use in personalized medicine could be a reality in the future.

Personalized medicine is arising as a tool to improve our understanding of disease and to develop unique therapeutic strategies based on individual pathological parameters. In this field, the use of nanotechnological devices and tools able to monitor biological biomarkers or quantify molecular profiles, will play a crucial role in its expansion and ultimately in the comprehension of human pathology [12]. In fact, several primary human cell models derived from patients are being used as unique models of disease [13]. These models enable to study the pathology and test therapeutic candidates, serving as a unique platform to understand and determine patient response to specific treatments. In order to further advance in this field, nanotechnology tools must be assayed and characterized in these models, that differ greatly from human cell lines. Human primary cells are directly isolated from tissues, including blood and bone marrow, and cultured in an artificial environment. Compared to cell lines, primary cell cultures provide more biologically relevant data but they usually have a limited lifespan. To overcome this drawback, some authors have developed a method to immortalize those primary cells, such as lymphocytes from patients, so they can be cultured as an immortalized cell line derived from a specific patient [14].

One of the fields in which a significant development in quantitative molecular profiling has been made is oncology. The possibility of tumor removal or biopsy in lab analysis has enabled an in depth analysis of tumoral mechanism and accurate diagnosis with the use of QDs [8]. Their capabilities allow molecular profiling through multi-target single cell imaging that results in an accurate quantification of tumor heterogeneity [9,15].

However, another more challenging line of research comprehends disorders of the central nervous system (CNS). Substantially limited to the possibility of biopsies and restricted to the study of postmortem samples, for many years the study of the CNS pathologies in humans has been hampered [16]. The improved characterization techniques that offer nanomaterials, and CdSe QDs in particular, would be a great advance in the understanding of these diseases using cells derived from patients. Amyotrophic lateral sclerosis (ALS) is a fatal neurodegenerative disease characterized by a progressive deterioration of upper and lower motor neurons that yields in a progressive paralysis. Some studies have confirmed the development of protein inclusions, mainly composed by TDP-43, in the cytosol of patients, that have a role in the disease progression. However, the mechanism underlying selective motor neuron death still remains an essential question [17].

Utilizing these methodologies, great efforts have been made to develop new models to study these diseases in human cells including neurons derived from iPSCs, fibroblasts and lymphoblasts. These models are demonstrating their ability to accurately recapitulate pathological patterns of neural diseases and are ideal tools to test therapeutic strategies [13]. However, the use of nanotechnological tools in these improved cellular models is scarce and a proper characterization is needed in order to understand the potential of luminescent particles including CdSe QDs in characterizing pathology individually and track therapeutic action.

In this work we characterize several commercially available QDs and their conjugates and monitor their behavior in the neural cell line SH-SY5Y and in fibroblast and lymphoblast cultures derived from ALS patients.

## 2. Materials and Methods

### 2.1. QD Bioconjugation

Amine-functionalized PEG-coated QDs with emission peaks centered at 520, 630 (CdSe/ZnS core-shell type quantum dots, Sigma-Aldrich, St. Louis, MO, USA), 605 and 655 nm (QDot ITK amino (PEG) QDs, Invitrogen, Waltham, MA, USA) were used for the synthesis of QD bioconjugates. Prior to the bioconjugation, QD stocks were centrifuged at 2000× *g* for 1 min at room temperature (RT) to remove potential QD aggregates. Then, QDs were activated with bifunctional BS3 crosslinker (bis(sulfosuccinimidyl)suberate, S5799 Sigma-Aldrich). 25 μL of 8 μM QDs stock (or 50 μL of 4 μM QDs stock) were mixed with 4 μL 50mM BS3 in 10 μL phosphate buffered saline (PBS, pH 7.8) and water, obtaining a 100 μL of 2μM QD solution in PBS with around 1000 molar excess of BS3. The mixture was incubated for 30 min at RT. Activated QDs were purified using a NAP-5 column (17085301, GE Healthcare, Boston, MA USA) pre-equilibrated with PBS, in order to remove the excess of BS3. A handheld UV lamp was used to track and collect around 500 μL QDs that were concentrated down to 40–50 μL using 100 kDa MWCO filters (UFC510024, Millipore, Burlington, MA, USA) by centrifugation at 7000× *g* for 7 min at RT. For the second step, collected QDs were incubated with 100 μL of 5mg/mL of SpA (protein A from *Staphylococcus aureus*, P6031 Sigma-Aldrich) or PG (protein G from *Streptococcus* spp., 0862 Sigma Aldrich) solution in PBS overnight at RT. Covalently-linked QDs were purified by centrifugation at 7000× *g* for 7 min at RT with an Amicon Ultra 100 kDa MWCO centrifugal filter (Millipore) at least six times. A spectrophotometer (Ultrospec 2100, Amersham Bioscience, Buckinghamshire, UK) was used to register QD-SpA or QD-PG bioconjugates absorption spectra and to calculate its final concentration. QDs bioconjugates were stored at 1 μM at 4 °C.

### 2.2. Transmission Electron Microscopy

A diluted solution of QDs or QD bioconjugates (0.2–0.5 μM) was dropped onto 400 Mesh copper-carbon grids and then dried at environmental conditions. Samples were stained with uranyl acetate 0.5% prior to their observation. Images were acquired using a JEM-1230 transmission electron microscope (JEOL, Peabody, MA, USA) operated at 100 kV. At least 10 different locations on the TEM grid were examined. Particle size was determine using Image J software (Bethesda, MD, USA). The diameter was calculated considering perfectly spherical shape of at least 50 different particles.

### 2.3. Dynamic Light Scattering

Dynamic light scattering (DLS) measurements were taken with a DynaPro MS/X instrument (Protein Solutions, Piscataway, NJ, USA) using a 90° light scattering cuvette. Prior to measure, QD samples were diluted to 0.1 μM in filtered PBS and then they were centrifuged at 12,000× *g* for 10 min. A total of 40 measurements of 10 s per sample were collected at 25 °C. The hydrodynamic radius was calculated with Dynamics V6 Software (Wyatt Technology Corporation, Santa Barbara, CA, USA) and data were represented as radius (nm) (x axis) versus % light intensity (y axis).

### 2.4. Electrophoretic Mobility

Agarose gel electrophoresis experiments were performed using 1.5% agarose gels with pH = 8.8 Tris acetate EDTA (TAE) running buffer and run for 30 min at 110 V. QDs and QD bioconjugates solution (5 μL, 0.25 μM) were mixed with 1 μL of loading buffer (G2526, Sigma-Aldrich) and loaded onto the gel. Images were taken using a Chemidoc Imaging System (Bio-Rad, Hercules, CA, USA).

### 2.5. Neuronal Cell Line Culture

Human SH-SY5Y cells were cultured in Dubelcco’s Modified Eagle Medium (DMEM, Gibco, Waltham, MA, USA) supplemented with 10% fetal bovine serum (FBS) and 1% penicillin-streptomycin and maintained in a humidified 5% CO_2_ in an incubator at 37 °C. Cells (7.5 × 10^4^) were grown in 24-well plate onto a previously sterilized glass-cover.

### 2.6. Amyotrophic Lateral Sclerosis (ALS) Patients

All study protocols were approved by the Hospital Doce de Octubre and the Spanish Council of Higher Research Institutional Review Board and in accordance with National and European Union Guidelines. All patients were diagnosed by applying the revised El Escorial criteria [18].

#### 2.6.1. Skin Fibroblast Cultures

Human skin fibroblasts were obtained from the dorsal region of the upper arm of an ALS patient. Fibroblasts from the biopsy specimens were cultured in DMEM supplemented with 10% FBS and 1% penicillin-streptomycin and maintained in a humidified 5% CO_2_ in an incubator at 37 °C. Cells (1.5 × 10^3^) were grown in 24-well plate onto a previously sterilized glass-cover. Cells cultures with a passage number greater than 12 were not considered for experiments.

#### 2.6.2. Lymphoblastic Cell Lines

Peripheral blood samples of ALS patients were collected after written informed consent to establish the lymphoblastoid cell lines as previously described, [13,19] by infecting peripheral blood lymphocytes with the Epstein Barr virus (EBV). Lymphoblastoid cell lines were grown in suspension in T flask in RPMI-1640 medium containing 2 mM L-glutamine, 10% FBS and 1% penicillin-streptomycin and maintained in a humidified 5% CO_2_ incubator at 37 °C.

### 2.7. Immunofluorescence

Cells were fixed with 4% paraformaldehyde (PFA) for 15 min at 37 °C. In order to improve intracellular access, PFA was supplemented with 0.05% of Triton-X-100 10% (93443, Sigma-Aldrich). For immunofluorescence (IF) staining procedure, up to three different permeabilization protocols were used: (a) cells were permeabilized with 0.25% Triton X-100 for 20 min and then rinsed three times. (b) In order to improve QD nuclear penetration, cells were treated with 2% DTAC (*n*-dodecyltrimethylammonium chloride, Sigma-Aldrich) for 20 min, rinsed three times with PBS, followed by 0.25% Triton X-100 for 5 min and washed three times with PBS. (c) Optionally, cells were also incubated with proteinase K (PK) (10–50 μg/mL in 1-2% SDS respectively) for 45 min to 1h at 37 °C. Once cells were permeabilized, they were blocked with 2% bovine serum albumin (BSA, 10735078001, Sigma-Aldrich) and 0.1% casein (C4765, Sigma-Aldrich) for 30 min at RT.

For a two-step IF, cells were incubated with primary antibodies for 1 h at 37 °C, monoclonal antibody anti-Histone H1 (SC-8030, Santa Cruz Biotechnology, Dallas, TX, USA), anti-α-Tubulin (SC-23948, Santa Cruz Biotechnology), anti-GAPDH (10494-1-AP, Proteintech, Manchester, UK), anti-TDP-43 (60019-2-Ig, Proteintech) and anti-phospho TDP-43 (22309-1-AP, Proteintech) were used in 6% BSA. After several washes with PBS, cells were incubated with goat anti-mouse QD655 or QD605, or goat anti-rabbit QD565 (1:50–1:100, Thermo Fisher Scientific, Waltham, MA, USA) in 6% BSA for 2 h at RT. For classical IF staining, cells were incubated with Alexa Fluor 488 or 568 secondary antibody conjugates (1:600–1:1000, 115-546-062, Jackson Immuno Research West Grove, PA, USA and A11011, Invitrogen) for 1 h at 37 °C. For multiplexing studies, a sequential based IF was performed. Each staining cycle was consisted of (i) blocking, (ii) staining with primary antibodies from different animal species, and iii) staining with QD-Ab2. For single-step IF, QD-Ab probes (QD-SpA-Ab or QD-PG-Ab) were prepared by incubating 6 μL of 1 μM QD-SpA or QD-PG with 1.5 μL of 0.2 mg/mL primary antibody for 1 h at RT. Each QD-Ab probe was prepared in a separate tube and then mixed and diluted in 6% BSA to a final volume of 300 μL. Cells were incubated with QD-Ab probes mixture for 2 h at RT.

Cell nuclei were stained using HCS NuclearMask Deep Red (1:250, H10294 Thermo Fisher Scientific) and cells were washed twice with 1% BSA and 0.1% casein and rinsed with PBS three times. Finally glass coverslips were mounted onto Fluoromount Mounting Medium (F4680, Sigma-Aldrich) and were stored at 4 °C. In both protocols, negative controls were performed without primary antibody.

### 2.8. Image Adquisition

Images were obtained using a confocal laser scanning microscopy (CLSM) Leica TCS SP5 with a 63x oil immersion objective. QDs, Alexa 488 and HCS NuclearMask were excited with different lasers at 405, 488 and 633 nm respectively and their individual emissions were collected at 500–550 for Alexa488, 525–545 for QD520, 555–575 for QD565, 595–615 for QD605, 645–660 for QD655 and 660–720 for HCS NuclearMask.

## 3. Results and Discussion

### 3.1. QDs Characterization

In order to obtain an extensive characterization of QD-Ab tools to use in human cell models, we purchased commercially available QDs including amine functionalized QDs and secondary antibody conjugated QDs from different vendors. QD size, charge and its emission spectra were determined using different methodologies. This characterization helps monitoring the lack of homogeneity and reproducibility between commercially available QDs batches, and therefore provides valuable information to analyze biological results.

**Figure 1:**
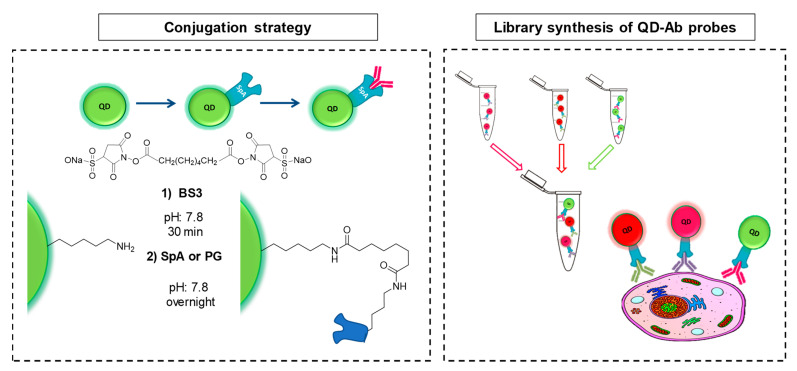
Bioconjugation strategy of QD-SpA or QD-PG and shematic illustration of the technology.

Amine functionalized PEG coated QDs were purchased from two different commercial sources and were covalently conjugated to adaptor proteins A (SpA) and G (PG) to bind to primary antibodies. This conjugation technique enables the use of very little amount of primary antibody and represents an advantage to direct covalent conjugation that requires greater quantity of biomolecule. Shortly, amine-funtionalized QDs were coupled to SpA or PG with the amine-to-amine crosslinker BS3 through two amide bonds. Then, QD bioconjugates were incubated individually with a primary antibody (ratio 1:3) at RT for 1 h to form QD-Ab probes (Figure 1).

Commercially available secondary antibody linked QDs were used to compare results with the conjugates generated in the laboratory.

#### 3.1.1. Transmission Electron Microscopy

**Figure 2:**
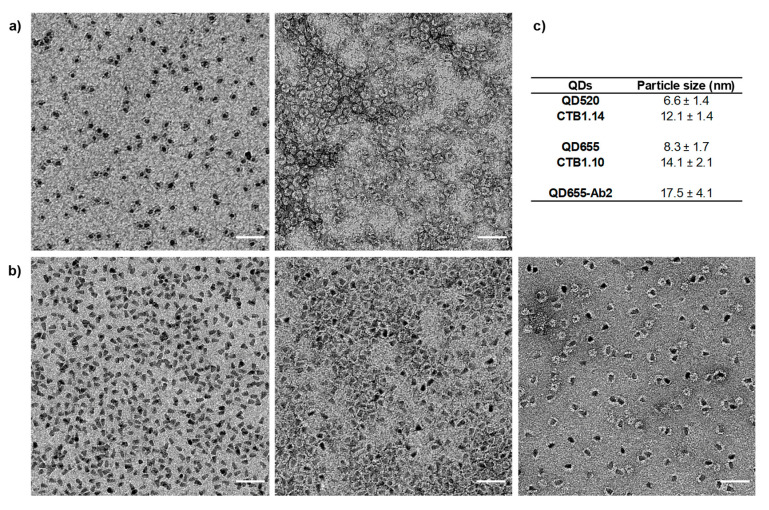
Transmission electron microscopy images of PEG-coated QDs, unconjugated, conjugated to SpA and commercially available QDs conjugated to secondary antibodies. (**a**) Unconjugated PEG coated QD520 (**left**) and bioconjugated QD520 to SpA, CTB1.14 (**right**). (**b**) Unconjugated PEG coated QD655 (**left**), bioconjugated QD655 to SpA, CTB1.10 (**center**) and commercially available QDs conjugate to secondary antibody (**right**). (**c**) Size of the different QDs analyzed. Scale bar 50 nm.

Two different sized amine functionalized PEG-QDs from Sigma-Aldrich and Invitrogen were analyzed by transmission electron microscopy (TEM). Size differences between unconjugated QD520 and QD655 were found with an average diameter of 6.6 ± 1.4 nm and 8.32 ± 1.7 nm respectively (Figure 2). These data are consistent with the size-dependent color tunability of QDs, since larger QDs emit in the red, while smaller QDs fluoresce in the blue [7]. Regarding the shape of the commercially available QDs, the 520 emitting nanoparticles presented and oval shape and the 655 QDs presented a sharper morphology. After covalent conjugation with adaptor protein A, both QDs, QD520 conjugate (CTB1.14) and QD655 conjugate (CTB1.10), exhibited a slight increase in their size, 12.1 ± 1.4 nm and 14.1 ± 2.1 nm respectively (Figure 2).

Furthermore, a distinct staining was observed. While QDs are stained in black, QD bioconjugates presented a white ring around the black dot. This confirmed the successful conjugation with adaptor proteins that are stained in white after uranyl acetate treatment. Size and morphology of commercially available secondary antibody QDs (QD-Ab2) were also analyzed by TEM (Figure 2). As expected, QD-Ab2 present a white ring around the black dot and an average size significantly larger (17.5 ± 4.1) and more heterogeneous (Figure S1). This increase in size compared to QD-SpA is mostly observed in the white ring around the nanoparticles, that was of approximately 6.0 nm (versus 3.0 nm for SpA) and not as uniform as in the SpA-QDs conjugated in the laboratory.

#### 3.1.2. Dynamic Light Scattering

**Figure 3:**
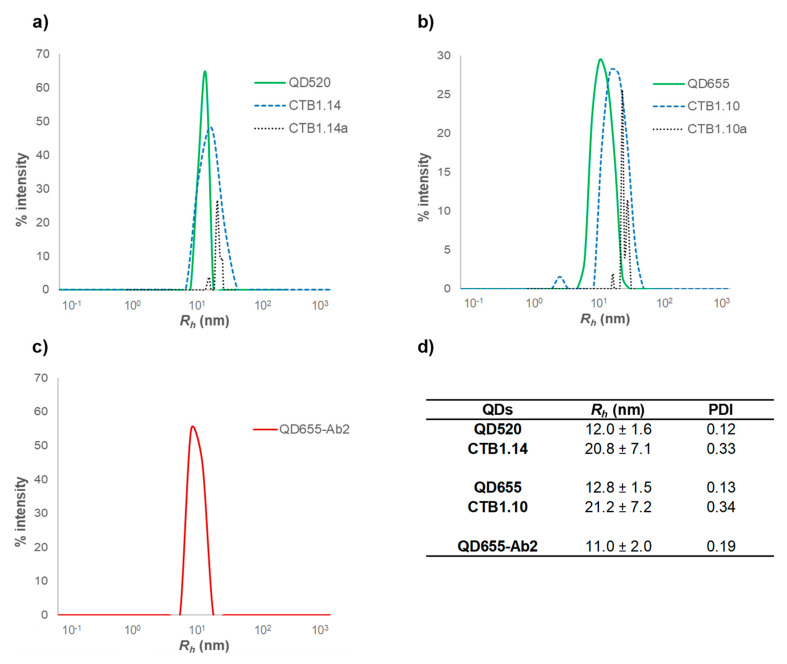
Size distribution of QDs, QD bioconjugates and QD-Ab2 obtained by DLS. (**a**) QD520 from Sigma-Aldrich, its protein A conjugate CTB1.14 and TDP-43 antibody conjugate CTB1.14a. (**b**) QD655 from Invitrogen, its protein A conjugate CTB1.10a and TDP-43 antibody conjugate CTB1.10a. (**c**) QD655-Ab2 from Invitrogen. (**d**) *R_h_* and PDI of QDs analyzed.

Dynamic light scattering (DLS) determines the size distribution profile of particles in suspension and it is a complementary technique to characterize the hydrodynamic size of nanoparticles [20]. Amine functionalized QD520 and QD655 exhibited a single peak with a hydrodynamic radius (*R_h_*) of 12.0 ± 1.6 and 12.8 ± 1.5 nm (green line) which increased up to 20.8 ± 7.1 and 21.2 ± 7.2 when conjugated to SpA (blue line) and up to 61.5 ± 8.0 and 82.8 ± 31.9 nm for the QD-SpA-Ab probes respectively (black line) (Figure 3). A second peak was observed for QD-SpA-Ab conjugates with a *R_h_* of 24.1 ± 9.3 and 21.0 ± 5.9 nm, corresponding to single QD-SpA that did not bind the Ab.

Moreover, significant differences were found regarding the PDI, which indicates the homogeneity of a particle solution. Values less than 0.15 may show a monodisperse particle suspension with a single size of QDs, while values higher than 0.15 indicate a heterogeneity particle solution with more than one population. In this sense, only unconjugated QDs showed a PDI less than 0.15. DLS results show that *R_h_* values are larger than the geometric radius of the QD conjugates measured by TEM [21] and also confirm the size difference between QD520 and QD655 and the successful bioconjugation of QDs to SpA and antibodies. Differences in the *R_h_* between unconjugated QDs, QD-SpA and QD-Ab2 conjugates are in agreement with previously reported results which sustain that the hydrodynamic size of commercial QDs vary substantially in function of the surface functionalization [22,23,24,25].

#### 3.1.3. Agarose Gel Electrophoresis

**Figure 4:**
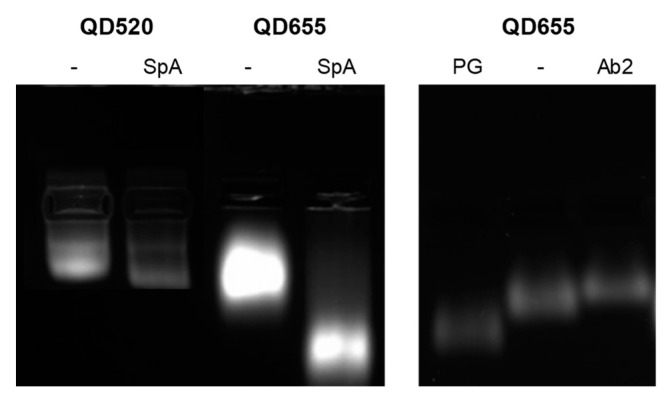
Electrophoretic mobility of unconjugated QDs, QD bioconjugates and commercially available QD-Ab2.

Electrophoretic mobility gives particle information both for the size and charge of QDs, allowing to confirm the conjugation of QDs to adaptor proteins or antibodies. 1.5% agarose gels were prepared and 5 μL of QDs or conjugates at 0.25 μM concentration were mixed with loading buffer and loaded in the wells. The gels were covered with TAE buffer at pH 8.8 and a current of 110 V was applied during 30 min. As it can be seen from Figure 4, all QD shifted from the original wells towards the positive end. When QDs were conjugated with adaptor proteins (SpA or PG) their mobility was higher than the amino functionalized original QDs in all cases. Adaptor proteins (SpA and PG) have a high percentage of acid amino acids in its structure [26], having an isoelectric point of 5.10 and 4.19 respectively [27,28]. Therefore, QD bioconjugates will show a higher number of negative charges due to those deprotonated amino acids at pH 8.8. Although conjugates are larger in size compared to the unconjugated nanoparticles it was observed how the difference in the charge added by the adaptor proteins determined the differential electrophoretic mobility. Therefore, this characterization will allow to confirm a successful conjugation and also check for batch reproducibility and nanoparticle homogeneity (Figure S2). QDs with secondary antibody also presented a shift towards the positive end of the gel, with a slightly less mobility than amine functionalized unconjugated QDs, probably due to their larger size (Figure 4).

#### 3.1.4. Spectral Characterization

**Figure 5:**
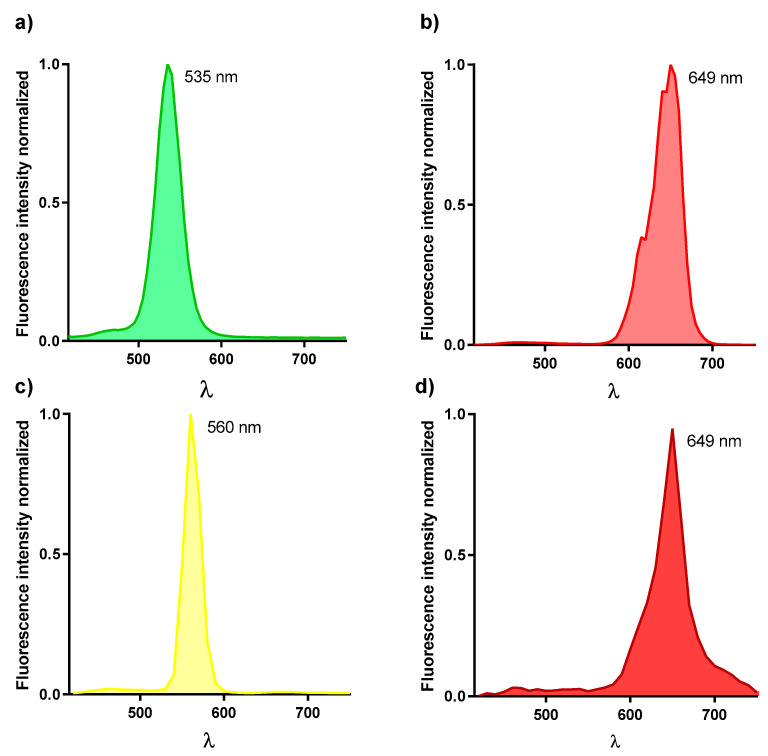
Spectral characterization of QDs. Emission spectrum of QDs was determined with a confocal microscope. Individual signals of QDs emission peaks were recorded. (**a**) QD520 (**b**) QD655 (**c**) QD565-Ab2 and (**d**) QD655-Ab2, and no spectral crosstalk between QD channels was observed.

QDs are characterized by significant narrow emission spectra, about 20–30 nm wide. In order to assure this and their maximum emission wavelength, their emission spectra was determined with a confocal microscope. A QD-immunoassay labelling **α-**tubulin (an ubicous protein) [29] in SH-SY5Y cells was performed with CTB1.22a (QD520-SpA-**α-**Tubulin Ab), CTB1.24a (QD655-SpA-**α-**Tubulin Ab) or QD-Ab2 conjugates. The emission spectra were recorded from the overall z planes. Maximum emission wavelengths were displaced, 15 nm for QD520, 6 nm for QD655 and QD655-Ab2, and 5 nm for QD565-Ab2 and therefore emission collection needed to be rearranged (Figure 5).

As expected, red emitting QDs presented wider emission spectra comparing QDs from the same commercial source. However, when comparing different commercial sources, green emitting QDs had a similar emission bandwidth than red emitting QDs from a different source.

The accuracy of the emission spectra of QDs, is critical for multiplexing experiments since QDs with emissions centered at close wavelengths are used [30]. In this sense, this type of characterization is required before performing multiplex studies to avoid cross-contamination.

### 3.2. Immunoassay Labelling

After the characterization, we performed QD-immunoassays in different cellular cultures including human primary cells in order to monitor any differences among QD probes and staining performance in cells derived from patients vs. commercial cell lines. In general, one of the main discrepancies found in the literature for staining with QDs is their ability for nuclear penetration and therefore, the staining of nuclear targets [25,31]. Here we utilized standard nuclear (Histone H1) and cytosolic (**α-**tubulin, GAPDH) proteins to test the nuclear penetration of different QD conjugates in cell lines, primary human cells and cellular models derived from patients.

#### 3.2.1. SH-SY5Y

**Figure 6:**
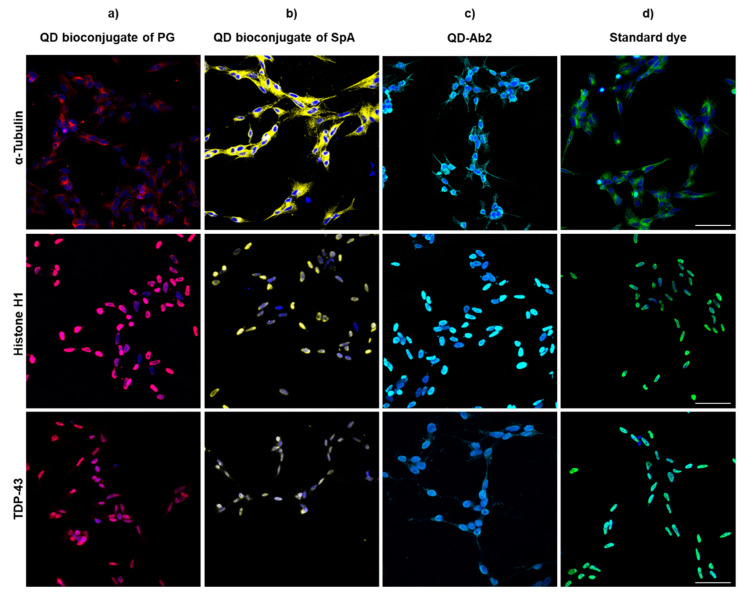
Staining of different commercially available QD conjugates and classic IF. **α-**Tubulin, Histone H1 and TDP-43 were stained in a one-step protocol with (**a**) QD655 bioconjugate of PG incubated with primary antibody (**b**) QD605 bioconjugate of SpA incubated with primary antibody; or in a two-step protocol with (**c**) QD655-Ab2 after primary antibody incubation and (**d**) standard dye after primary antibody incubation. To label cytoplasmic targets Triton 0.25% was used as permeabilization agents in all cases. For nuclear targets further permeabilization was needed using Triton 0.25% and DTAC 2% for QD bioconjugates and PK 30 μg/mL for QD-Ab2. A standard dye was used as control of no protein degradation. Nuclei were stained with HCS Nuclear Mask Deep Red. Scale bar: 20 μm.

Different commercially available QDs conjugated to secondary antibody (QD-Ab2) and QD bioconjugates (QD-SpA or QD-PG) were used to label reference proteins such as histone H1 or α-tubulin in order to determine nuclear penetration of the conjugates. In addition, a key target in neurodegenerative diseases, TDP-43, was also labeled [32]. This protein is encountered physiologically in the nucleus and aggregated in the cytosol under pathological conditions in neurodegenerative diseases [33] and therefore, its staining in cells derived from patients has enabled an accurate pathological characterization. In order to contrast the images obtained and validate QDs specific labelling, a classic immunofluorescence (IF) was performed. QD bioconjugates with adaptor proteins (SpA or PG) were first incubated with primary antibodies to form a QD probe and directly incubated in a one-step protocol. A two-step protocol with primary antibody and secondary antibody labelled with QD or standard dye was also used. As seen in Figure 6, all three targets were successfully labelled with the different QDs achieving a similar staining to classic IF. However, in all cases, to achieve nuclear staining several permeabilization protocols were performed since standard IF conditions did not yield nuclear staining with QDs (Figure S3).

Different detergents and permeants including Triton, DTAC and proteinase K (PK) were used to optimize histone H1 labelling in SH-SY5Y cells. Finally, to obtain a nuclear staining, the conditions needed to be used were DTAC 2% + 0.25% Triton for the conjugates with adaptor proteins and a further PK (30 μg/mL) protocol for the commercially available QD-Ab2 probes for this particular cell line. In these conditions both QD-SpA-Ab, QD-PG-Ab and QD-Ab2 were capable of staining the nucleus. It is also worth mentioning that while the probe ratio of the commercially available QD-Ab2 is given by the vendor, it must be optimized for the prepared QD-Spa-Ab and QD-PG-Ab conjugates in order to obtain high quality images.

#### 3.2.2. Primary Human Fibroblasts

**Figure 7:**
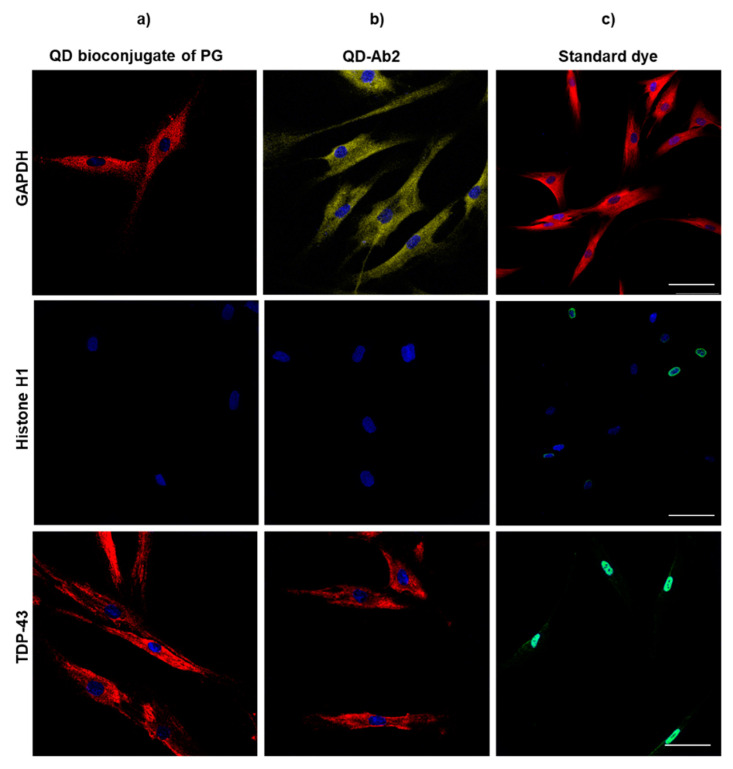
GAPDH, Histone H1 and TDP-43 labelling with different commercially available QD conjugates and classic IF in fibroblasts from an ALS patient. GAPDH, Histone H1 and TDP-43 were stained in a one-step protocol with (**a**) QD655 bioconjugate of PG incubated with primary antibody or in a two-step protocol with (**b**) QD655-Ab2 after primary antibody incubation and (**c**) standard dye after primary antibody incubation. For labelling cytoplasmic targets Triton 0.25% was used as permeabilization reagent in all cases. No nuclear staining was achieved with QD bioconjugates even using further permabilization with PK 30 μg/mL. Nuclei were stained with HCS Nuclear Mask Deep Red. Scale bar: 20 μm.

After having optimized cytoplasmic and nuclei labeling with the different QD probes in the neuroblastoma cell line, amyotrophic lateral sclerosis (ALS) derived fibroblasts were used. Following the same protocols established in the neural cell line, it was observed that in these primary human cells, QDs were not able to label histone H1, probably due to the inability to permeate the nucleus in these human primary cells. Figure 7 shows successful labeling of cytoplasmic target GAPDH and cytoplasmic TDP-43 but histone H1 or nuclear TDP-43 staining was never observed even with the most aggressive permeabilization conditions. This finding is in agreement with other studies in which it has been observed how nanoparticle permeability in live neural cell lines is significantly higher than in primary cells [34,35].

Although working with fixed cells enables the use of several permeants in order to achieve staining it must be noted that even with harsh permeabilization conditions nuclear staining may not be acquired. Additionally, when using these strong agents, cellular membranes are disrupted and it has been observed how these techniques could lead to artifacts or artificial changes in the cells (Figure S4) [30]. However, permeability must always be checked for the specific nanomaterial used since it has also been observed how not only size but also charge or coating can have an effect in the permeabilization of nanoparticles [36].

#### 3.2.3. Immortalized Human Lymphocytes

Next, immortalized lymphocytes derived from ALS patients were examined and stained for the same targets with the different QD probes. Lymphoblasts have been suggested to recapitulate neural pathology, specifically in neurodegenerative diseases such as ALS [13] or frontotemporal dementia [37]. Immortalized with the Epstein-Barr Virus [14], lymphoblasts can divide indefinitely in cell culture conditions. This procedure enables to have a significant source of cells from patients and offer a unique platform to study the disease individually. Additionally, they are an ideal model to examine the effect of potential drugs and determine patient-specific therapeutic response. In highly heterogeneous diseases such as ALS, it is a powerful tool to elucidate the complex pathology in different genetic and sporadic cases of this rare neurodegenerative disease.

**Figure 8:**
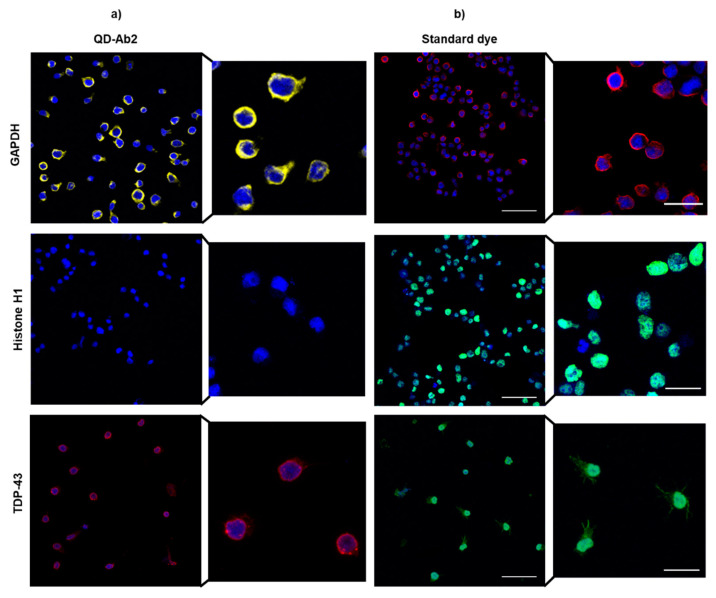
ALS lymphoblasts were labelled with GAPDH, Histone H1 and TDP-43 with (**a**) QD-Ab2 and (**b**) standard dye for multiple comparison. No nuclear staining was observed with QD-Ab2 even using PK 30 μg/mL. GAPDH and cytoplasmic TDP-43 were stained using Triton 0.25% as permeabilization reagent. Nuclei were stained with HCS Nuclear Mask Deep Red. Scale bar: 20 μm.

Similarly to the IF assay in fibroblasts, QD-Ab2 commercially available conjugates were able to successfully stain cytosolic proteins with high fluorescence stability. However, the staining of nuclear proteins was not achieved even employing several permeabilization agents (Figure 8). Additionally, staining with the adaptor protein conjugates was not observed. In this case, these probes synthesized in the lab were not suitable for staining in patient derived lymphoblasts.

### 3.3. Multiplexing in Lymphoblasts from Patients

**Figure 9:**
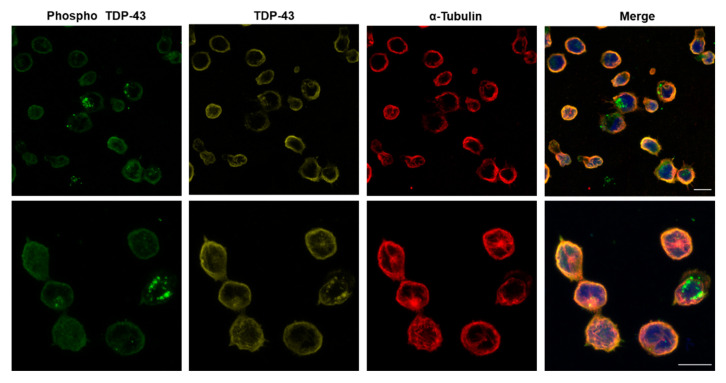
Phospho TDP-43, TDP-43 and **α-**Tubulin are simultaneously stained with QD-Ab2 emitting at 565, 605 and 655 nm, respectively in a sporadic ALS patient. Nuclei were stained with HCS Nuclear Mask Deep Red. Scale bar: 20 μm.

Finally, we performed multiplexing studies in lymphoblasts derived from ALS patients in order to assess the potential of this experiment to target key ALS proteins in patients cells with QD conjugates. As shown in Figure 9, QD-Ab2 conjugates were able to successfully stain **α-**tubulin (red), phospho TDP-43 (green) and TDP-43 (yellow) in ALS lymphoblasts. For cross validation, single staining of each target was performed and no fluorescence intensity was observed in the other channels. A negative control was also made with other QD-Ab2 from the same specimen and no crosstalk between different QDs was observed. Compared to single IF staining, similar patterns of staining were obtained (data not shown).

## 4. Conclusions

Here we report a comparative study of the use of commercially available CdSe QDs and their conjugates to perform IF in human neuroblastoma cell line SH-SY5Y and cells derived from ALS patients. Several techniques, including transmission electron microscopy (TEM), dynamic light scattering (DLS), and agarose electrophoresis were used to characterize the different probes. These techniques were also employed to monitor the successful bioconjugation of QDs to antibodies using two different adaptor proteins. From the IF experiments, several differences were observed in QD nuclear penetration depending on the specific QD conjugate used or the cellular model, suggesting the necessity to perform extensive characterization techniques and protocols to achieve specific staining. Finally, using these tools, we achieved a multiplexed staining of three targets on lymphoblasts derived from ALS patients which could be used to perform molecular profiles in these type of patients with QD nanoparticles.

## Data Availability

The data presented in this study are available on request from the corresponding author.

## Supplementary Materials

The following are available online at https://www.mdpi.com/2079-4991/11/3/671/s1, Figure S1: Size distribution of QDs, QD conjugate and QD-Ab2 measured by TEM. Figure S2. Electrophoretic mobility QD conjugates. Figure S3. Nuclear penetration of QDs in SH-SY5Y cells. Figure S4. QD staining with different permeabilization conditions.
