# Supplementary material for: CdSe Quantum Dots in Human Models Derived from ALS Patients: Characterization, Nuclear Penetration Studies and Multiplexing
Source: Nanomaterials (Basel). 2021 Mar 9;11(3):671. doi: 10.3390/nano11030671 (PMC7998605; doi:10.3390/nano11030671)
Supplement: Supplementary file 1 [file nanomaterials-11-00671-s001.pdf]

Supplementary File

CdSe Quantum Dots in Human Models Derived from ALS Patients: Characterization, Nuclear Penetration Studies and Multiplexing

Carlota Tosat-Bitrián [1], Alicia Avis-Bodas [1], Gracia Porras [1], Daniel Borrego-Hernández [2], Alberto García-Redondo [2], Angeles Martín-Requero [1,3] and Valle Palomo [1,3,*]

[1] Centro de Investigaciones Biológicas Margarita Salas CSIC, C/Ramiro de Maeztu 9, 28040 Madrid, Spain; carlota.tosat@cib.csic.es (C.T.-B.); aliciaavis97@gmail.com (A.A.-B.); graciapf@cib.csic.es (G.P.); amrequero@cib.csic.es (Á.M.-R.)

[2] Neurology Department, ALS Unit, CIBERER U-723, Health Research Institute, 28041 Madrid, Spain; dborregohernandez.imas12@h12o.es (D.B.-H.); mito@h12o.es (A.G.-R.)

[3] Centro de Investigación Biomédica en Red de Enfermedades Neurodegenerativas (CIBERNED), Instituto de Salud Carlos III, 28031 Madrid, Spain

* Correspondence: vpalomo@cib.csic.es

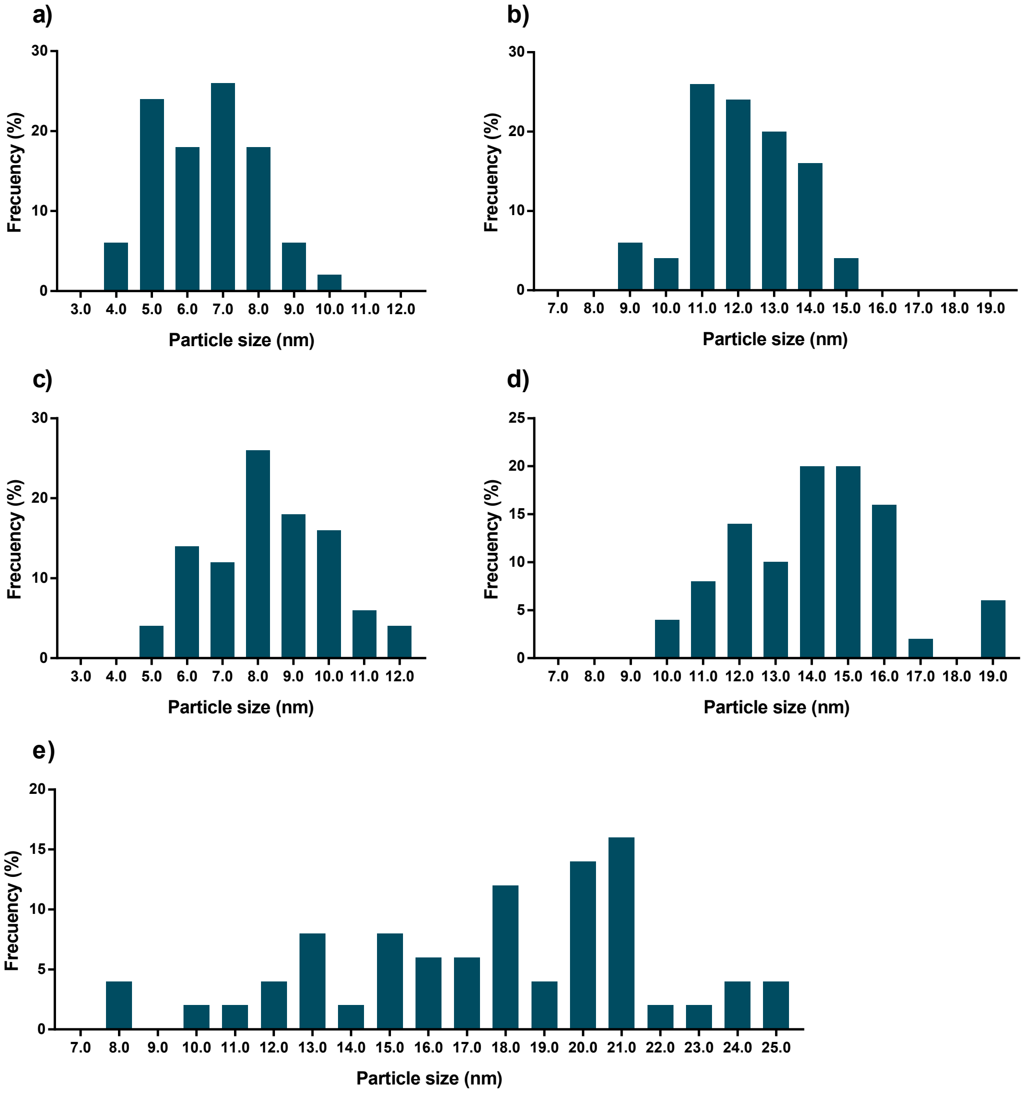

Figure S1. Size distribution of QDs, QD conjugates and QD-Ab2 measured by TEM. a) QD520, b) CTB1.14 (QD-SpA) c) QD655, d) CTB1.10 (QD-SpA) and e) QD655-Ab2.

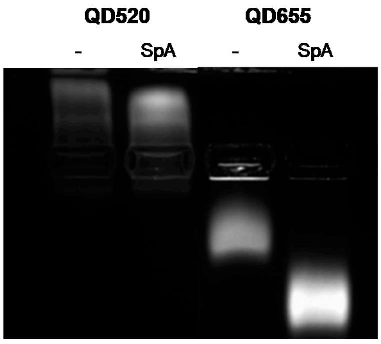

Figure S2. Electrophoretic mobility of unconjugated defective 520 QD and standard 655 QDs and their biocongujates with SpA. The defective QD520 and its bioconjugate to SpA migrate towards the negative pole.

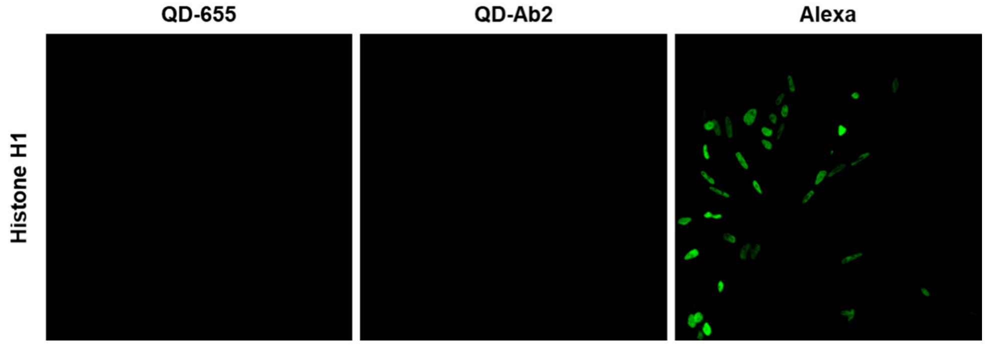

Figure S3. Histone labelling with QD655 bioconjugate, QD-Ab2 and Alexa fluorphore using as permeablization reagent Triton 0.25% for QD-655 and DTAC 2% + Triton 0.25% for QD-Ab2.

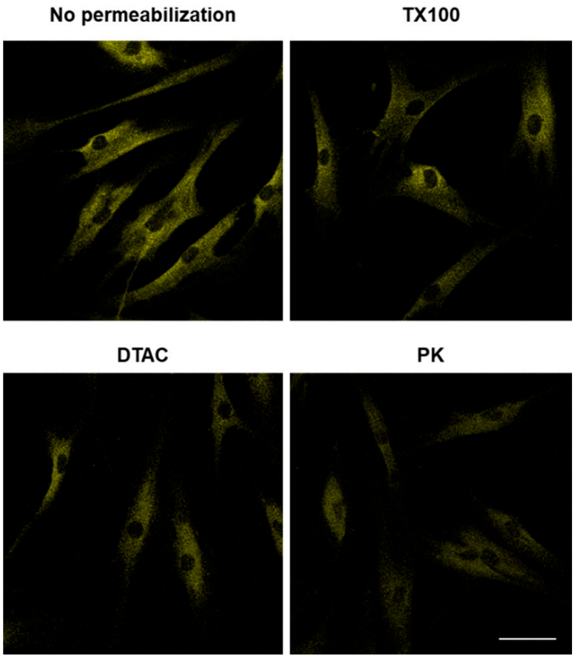

Figure S4. QD staining of GAPDH with different permeabilization conditions. Protein diggestion of cytosolic targets after treatment with stronger permabilization agents. Treatment with PK improves nuclear access but a degradation of cytoplasmatic targets is observed.
